# Noble Metals Deposited LaMnO_3_ Nanocomposites for Photocatalytic H_2_ Production

**DOI:** 10.3390/nano12172985

**Published:** 2022-08-29

**Authors:** Ahmed Hussain Jawhari, Nazim Hasan, Ibrahim Ali Radini, Katabathini Narasimharao, Maqsood Ahmad Malik

**Affiliations:** 1 Department of Chemistry, Faculty of Science, Jazan University, Jazan 45142, Saudi Arabia; 2 Chemistry Department, Faculty of Science, King Abdulaziz University, Jeddah 21589, Saudi Arabia

**Keywords:** LaMnO_3_, noble metals, green synthesis, photocatalysis, visible light, H_2_ production

## Abstract

Due to the growing demand for hydrogen, the photocatalytic hydrogen production from alcohols present an intriguing prospect as a potential source of low-cost renewable energy. The noble metals (Ag, Au, Pd and Pt) deposited LaMnO_3_ nanocomposites were synthesized by a non-conventional green bio-reduction method using aqueous lemon peel extract, which acts as both reducing and capping agent. The successful deposition of the noble metals on the surface of LaMnO_3_ was verified by using powder XRD, FTIR, TEM, N_2_-physisorption, DR UV-vis spectroscopy, and XPS techniques. The photocatalytic activity of the synthesized nanocomposites was tested for photocatalytic H_2_ production under visible light irradiation. Different photocatalytic reaction parameters such as reaction time, pH, catalyst mass and reaction temperature were investigated to optimize the reaction conditions for synthesized nanocomposites. Among the synthesized noble metal deposited LaMnO_3_ nanocomposites, the Pt-LaMnO_3_ nanocomposite offered superior activity for H_2_ production. The enhanced photocatalytic activity of the Pt-LaMnO_3_ was found as a result from low bandgap energy, high photoelectrons generation and enhanced charge separation due to deposition of Pt nanoparticles. The effective noble metal deposition delivers a new route for the development of plasmonic noble metal-LaMnO_3_ nanocomposites for photocatalytic reforming of aqueous methanol to hydrogen.

## 1. Introduction

Hydrogen (H_2_) energy, as a clean, efficient, non-toxic, and renewable energy, has diverse applications, to promises towards the future energy structure [1,2]. Hydrogen energy can meet the increasing demand for sustainable clean energy. In addition, hydrogen can be used to generate electricity without harmful emissions in industrial applications. It is anticipated to be the cleanest energy source and an answer to the energy crisis and associated climate change issues [3]. H_2_ has received greater attention as it also has storage option; it allows energy to be carried and converted when required [4]. Currently, metal hydrides are utilized for conventional hydrogen storage, but their system’s storage capacity is inadequate. Fuel cell-based hydrogen is a better alternative [5,6]. Due to its low carbon emissions and high potential efficiency, the H_2_ fuel cell system offers an alternative to internal combustion engines for automobiles [7]. Because of the varied environmental advantages, higher efficiency and potential market, these systems have gained more attention [8].

Biomass is an important source of H_2_ [9] and it is mainly produced by biomass gasification, but this process is limited to specific applications [10]. Water electrolysis is a well proven technology, which is the future of H_2_ production, basically because it relies on an inexhaustible source, such as water. However, it uses a lot of energy, and this is a serious concern [11]. Photocatalytic water splitting [12] and photocatalytic reforming of bio-alcohols [13] are two promising methods for the sustainable production of H_2_, and the second process combines simultaneous H_2_ production [14]. H_2_ can be produced on a large scale through numerous technical processes. Among the thermal and nonthermal processes, the steam-reforming process exhibited four times higher H_2_ productivity compared to the photocatalytic non-thermal process [15]. Due to the expensive nature of H_2_ storage and transportation, the green methanol has become a safe and efficient alternative to promote and develop H_2_ energy [16]. The conventional thermal methanol-reforming reaction to produce H_2_ is an endothermic reaction; the energy required for the reaction is mainly provided by furnace heat, therefore, it cannot be termed as renewable energy [17].

Solar photocatalytic H_2_ production is ideally the best pathway to convert solar energy to chemical energy. Different semiconductors such as TiO_2_, ZnO, g-C_3_N_4_, Ag_3_PO_4_ and ZrO_2_ have been predominantly studied for photocatalytic H_2_ production from aqueous alcohols [18,19,20]. More recently, lanthanum-based perovskites have been proposed as photocatalytic material owing to its characteristic properties such as narrow bandgap (Eg = 1.86–2.36 eV), high stability as well as their environmental friendliness [21]. The photocatalytic properties of lanthanum (La) -based perovskites can further be enhanced via deposition of different metal nanoparticles. The presence of metal nanoparticles enhances charge transfer and light captivation through local surface plasmon resonance (LSPR), improving catalytic performance. Wang et.al. synthesized porous La-Ti based perovskite (La_2_Ti_2_O_7_) using CTAB as a template reagent for degradation of the dye azophloxine [22]. Moreover, the photocatalytic performance of La-based perovskites was enhanced using multiple complex or layered perovskite oxides containing Ln. Verduzco et al. synthesized a layered perovskite oxide (Sr_2.7−x_Ca_x_Ln_0.3_Fe_2_O_7−δ_) and demonstrated its use towards degradation of methylene blue (MB) dye under solar and UV irradiation [23]. Varying the stoichiometry or doping of perovskite with a cation (Ca-doping), of different valence states, can change the electronic structure, affecting the electrical and optical properties [24]. Yang et al. immobilized LaFeO_3_ and Au nanoparticles on the Cu_2_O surface as a ternary composite photocatalyst for rhodamine B degradation [25]. Numerous reports have shown that incorporation or decoration of Ln based perovskites with other metals or semiconductors results in pronounced enhancement in the photocatalysis [26,27,28,29]. The deposition of noble metal nanoparticles on the surface of polar semiconductor or insulator particles can enhance the photocatalytic activity by harvesting visible light due to the LSPR phenomenon, while the metal-semiconductor interface efficiently separates the photogenerated electrons and holes [30].

The present study focuses on the synthesis and characterization of noble metals deposited LaMnO_3_ nanocomposites for photocatalytic H_2_ production. A novel green procedure is adopted for the synthesis of bulk and noble metal deposited LaMnO_3_ nanocomposites. The H_2_ production efficacy of the indigenously synthesized, noble metal containing LaMnO_3_ nanocomposites was evaluated and discussed in this report for the first time.

## 2. Experimental

### 2.1. Materials

All chemicals, including Lanthanum(III) nitrate hexahydrate (La(NO_3_)_3_·6H_2_O), Manganese(II) nitrate hydrate (Mn(NO_3_)_2_·4H_2_O), Silver nitrate (AgNO_3_), Palladium(II) chloride (PdCl_2_), Platinum(II) chloride (PtCl_2_) and gold(III) chloride (AuCl_3_) are of analytical grade and purchased from Sigma-Aldrich, St. Louis, MO, USA.

### 2.2. Preparation of Nanomaterials

#### 2.2.1. Synthesis of LaMnO_3_ by Green Solid-State Combustion Method

LaMnO_3_ nanocomposite was prepared by the green solid-state combustion method. First, a homogenous mixture of La(NO_3_)_3_ and Mn(NO_3_)_2_ was prepared by mixing 5.0 g of each metal precursor in an agate mortar and grounded using a pestle for 15 min at room temperature. To this homogenous precursor mixture, 20 g of fine, dried lemon peel powder was added and ground for another 30 min to acquire a perfect blend of the metal precursor and lemon peel powder. Then, the resultant powder was transferred into a ceramic crucible and calcined in a muffle furnace at 700 °C for 4 h. It was found that the mixture transformed into a fine, pitch-black powder suspended several times in double distilled water and absolute ethanol for washing; then, it was dried at 120 °C for 6 h in a vacuum oven.

#### 2.2.2. Deposition of Noble Metals

**Figure 1:**
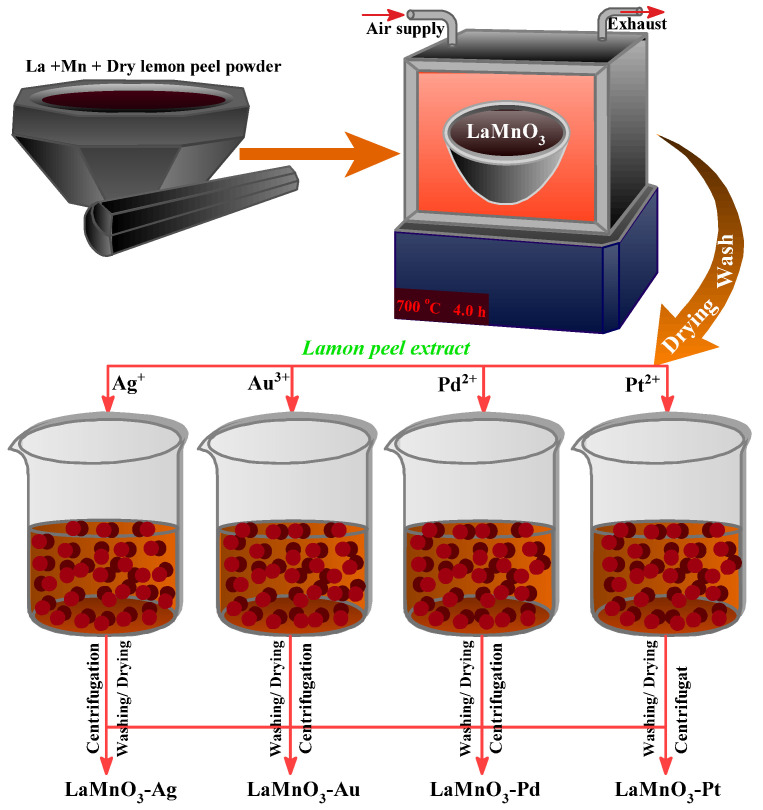
Schematic representation of the preparation of bulk and noble metal deposited LaMnO_3_ catalysts.

Deposition with noble metals is an efficient strategy to extrapolate materials’ photocatalytic and optical performance. The deposition of LaMnO_3_ nanocomposites was carried out by the green bio-reduction method using aqueous lemon extract solution as a reducing and capping agent. The lemon peel extract has the major compound limonene, which is believed to act as a reducing agent. The fresh lemon peel extract was prepared by dispersing 10 g of fine dried lemon peel powder in 250 mL of distilled water and heating at 80 °C for 1 hr. The mixture was filtered using Whatman No. 1 filter paper by vacuum filtration. In four different beakers, 2 g of LaMnO_3_ nanocomposites was dispersed in 10 mL of double distilled water under constant stirring. To each beaker, 10 mL of 0.15 M aqueous solution of Ag/Pt/Au/Pd salt was added, under vigorous stirring, followed by the addition of lemon extract (20 mL). The initial reduction of noble metals was observed from the appearance of color change. The noble metal loaded LaMnO_3_ nanocomposites were collected by centrifugation, and the acquired material was washed several times with double distilled water followed by drying at 90 °C for 5 h. Figure 1 gives the schematic representation of the nanocomposite preparation procedure.

### 2.3. Characterization of Synthesized Nanomaterials

The PANalytical XpertPro diffractometer (PANalytical Inc., Westborough, MA, USA) was used to collect the XRD patterns of the powders. Applying the Debye–Scherer equation allowed for the determination of the crystallite size of the nanocomposites that were formed. Fourier transform infrared spectroscopy (FTIR) analysis of as prepared nanocomposites was performed on a Bruker ALPHA II FT-IR (Bruker Optics GmbH & Co. Rosenheim, Germany) spectrometer. For the transmission electron microscopy (TEM) investigation of the nanocomposites, a JEOL 2100HT microscope equipped with a 200 kV accelerating voltage was utilized, and images were captured using a Gatan digital camera. The X-ray photoelectron spectra of the nanocomposites were gathered with the use of an instrument called Thermo Scientific Escalab 250 Xi XPS. The Al K X-rays used in the experiment had a spot size of 650 mm. We were able to rectify the peak shift that occurred as a result of charge compensation by employing the binding energy of C1s peak. The information was gathered by performing 10–30 scans with a step size of 0.1 eV, a dwell length of 200 ms, and a pass energy of 100 eV.

Experiments with N_2_-physisorption employing the Quantachrome ASiQ adsorption device were used to collect information regarding the textural qualities of the materials. The reflectance spectra of the samples were taken with a Thermo-Scientific evolution UV-vis spectrophotometer that was equipped with an integrating sphere in the wavelength range of 200–800 nm. This was performed so that the optical properties of the samples could be determined. The Kubelka–Munk method was utilized to determine the bandgap energy values of each sample. The Kubelka–Munk factor, denoted by the letter K, was calculated with the help of the following equation: K = (1 − R)^2^/2R, where R refers to the percent reflectance. After the wavelengths (nm) were converted into energy (E), a curve was constructed by plotting (K*E)^0.5^ against E to determine the relationship between the two variables. The bandgap energy, denoted in eV, was found at the point on the curve where the two slopes intersected.

### 2.4. Photocatalytic Reforming of Methanol to Hydrogen

Under the influence of an argon environment, photocatalytic reactions were carried out in the liquid phase inside of a Pyrex flask. To equilibrate any adsorption process and achieve a homogeneous catalyst suspension, the catalyst, which weighed 150 mg, was disseminated by stirring at 500 rpm in 120 mL of a 20 vol% methanolic aqueous solution at 25 °C for thirty minutes while maintaining complete darkness. After that, the reactor was purged of its contents, and a Xe lamp with 300 W of power was used to irradiate the reaction zone with a flux of roughly 125 mW cm^−2^ for an hour. Using a closed gas circulation, evolved gases were brought into the sample loop of the gas chromatograph. To perform the H_2_ analysis, a Varian 3300 gas chromatograph was utilized. This instrument was equipped with a thermal conductivity detector and a 2 m MS 13X column. The samples were analyzed periodically.

## 3. Results and Discussion

### 3.1. Characterization of LaMnO_3_ Nanocomposites

**Figure 2:**
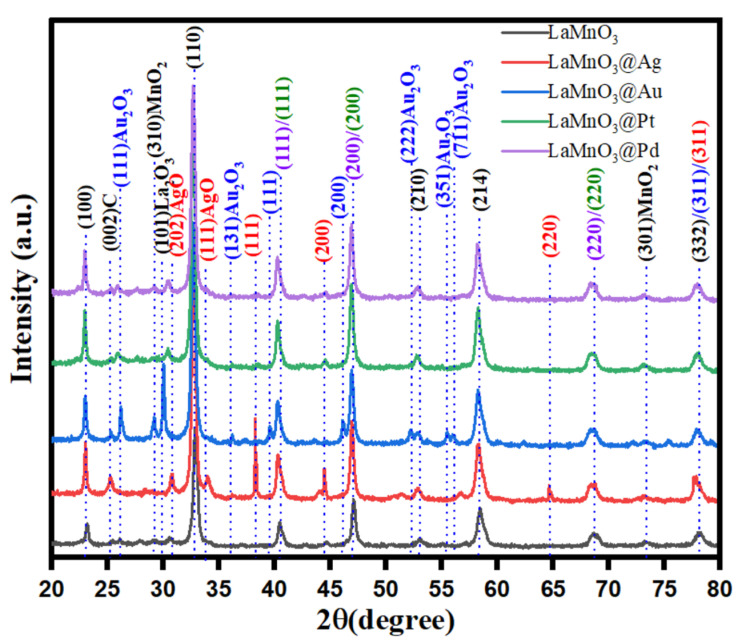
XRD patterns for the bulk and noble metal deposited LaMnO_3_ nanocomposites.

The X-ray diffraction (XRD) patterns for the synthesized samples were obtained to study their crystalline properties. The obtained patterns for bulk and the noble metal deposited LaMnO_3_ samples (Pt-LnMnO_3_, Pd-LnMnO_3_, Au-LnMnO_3_, and Ag-LnMnO_3_) are shown in Figure 2. The reflections at 2*θ* angles 33, 41, 58, 68 and 78° are indicative of the presence of crystalline LnMnO_3_ in all samples [31]. The characteristic reflections at 2*θ* angles of 38.12° (111), 44.27° (200), 64.42° (220), and 77.47° (311) for Ag (ICDD PDF file number 00-004-0783) [32], distinct peaks at 38.12, 44.27 and 64.42° were observed in case of Ag- LnMnO_3_ sample.

The XRD pattern of the Au-LnMnO_3_ sample exhibited additional reflections at 38.2° (111), 44.4° (200), 64.57° (220), and 77.54° (311) corresponding to the face-centered cubic structure of Au [ICDD PDF file number 00-004-0784]. The reflections at 39.6, 47.4 and 67.1° in case of Pt-LaMnO_3_ sample could be attributed to the reflections (111), (200), and (220) respectively, which are consistent with the face centered cubic (fcc) structure of Pt metal (JCPDS Card 04-0802]. The XRD pattern of Pd-LaMnO_3_ sample displays three peaks at 40.26, 45.78, and 68.67°, corresponding to (111), (200), and (220) planes, respectively. These can be indexed to the face-centered cubic (fcc) phase of Pd NPs [JCPDS ≠ 89-4897]. The observed XRD results clearly indicates the presence of noble metal crystallites in the synthesized samples.

**Figure 3:**
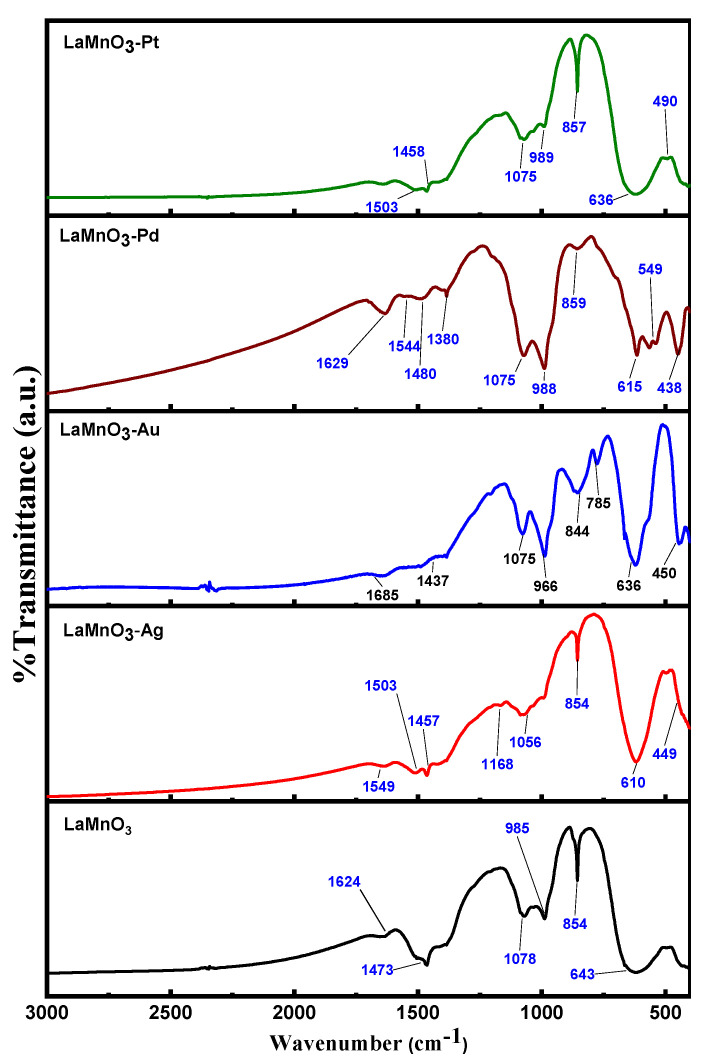
FT-IR spectra for the bulk and noble metal deposited LaMnO_3_ nanocomposites.

The FT-IR spectra for bulk and noble metals deposited LaMnO_3_ samples are shown in Figure 3. The FT-IR bands were observed at 643, 854, 985, 1078, 1473, and 1624 cm^−1^ for all the samples. It was previously reported that significant absorption bands were around 600, 820, 1100, 1380, 1450, and 1650 cm^−1^ for LaMnO_3_ nanoparticles. The major IR absorption band in the range of 615-643 cm^−1^ is due to the stretching mode for Mn-O-Mn bonds associated with the octahedron MnO_6_. The presence of this vibrational band, which is a characteristic of the ABO_3_ type perovskite [33], indicates the successful synthesis of LaMnO_3_ structure. The bands around 1624 cm^−1^ are assumed to correspond to bending vibrations of the N-H bonds (secondary amines); the bands around 1380 and 1450 cm^−1^ are produced by bending vibrations in the bonds N-O (nitrates) [34]. As observed from the FT-IR spectrum in Figure 3, the deposition of noble metals resulted a small shift in the band position.

**Figure 4:**
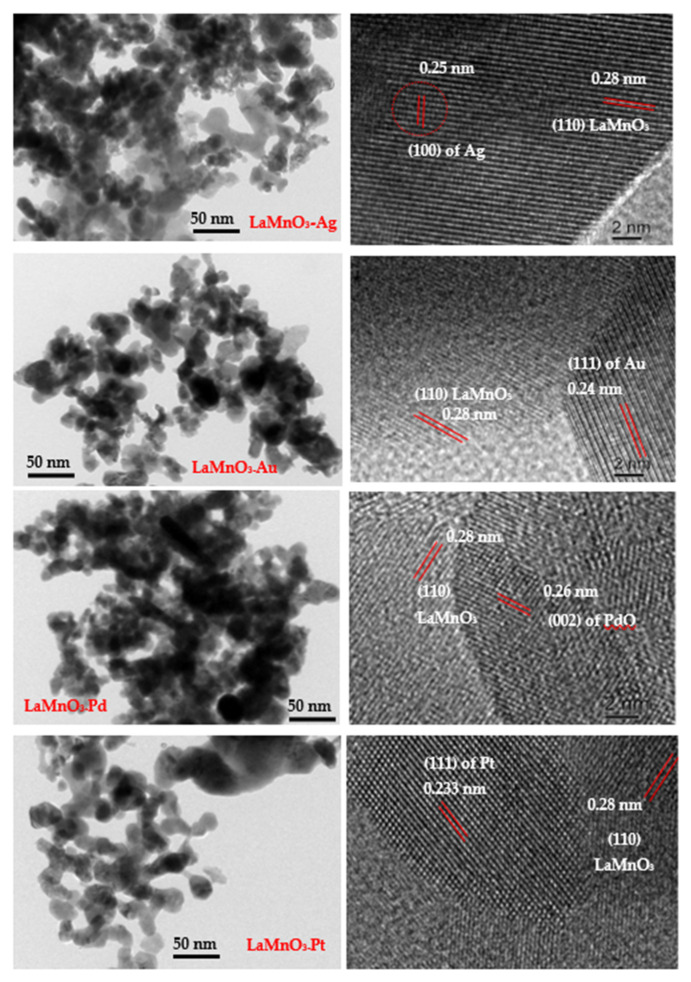
TEM and HRTEM images of the synthesized nanocomposites.

The morphology and particle sizes of different phases in the synthesized nanocomposites were studied using TEM analysis (Figure 4). As observed from the figure, the LaMnO_3_ nanoparticles with the sizes in the range of 10–25 nm was aggregated into clusters to form like nanorods with random distribution of particles. Apparently, the deposition of noble metals on the surface of LaMnO_3_ increased the agglomeration of particles probably due to usage of green bio-reduction using aqueous lemon extract. The TEM images of noble metal deposited samples clearly show dark nanoparticles of size in the range of 5–10 nm. The size variation in the noble metal nanoparticle size between the different noble metals was found to have no correlation with their atomic radius. It is probably because different metals possess different nucleation growth. The TEM images also show that all the samples exhibit meso to macro pore structure morphology as confirmed by the N_2_ adsorption isotherms of the samples (in later section). The meso and macro porosity is due to the random arrangement of the LaMnO_3_ particles. It was observed that Au, Ag and Pd containing samples possess large size pores, as shown by the TEM images. The surface nanoparticles and through-channels become obvious, which is due to the removal of different organic molecules (mainly citric acid) presented in the lemon extract during the thermal treatment. The HRTEM images clearly show LaMnO_3_ particles with a lattice-spacing distance of 0.28 nm all synthesized samples; this observation is in accordance with the lattice spacing of the (110) plane of LaMnO_3_ [35]. In addition, the presence of lattice fringes for metal and metal oxide nanoparticles (Ag, Au, Pd and Pt) appeared in the samples (Figure 4). These observations clearly corroborate the XRD analysis results.

**Figure 5:**
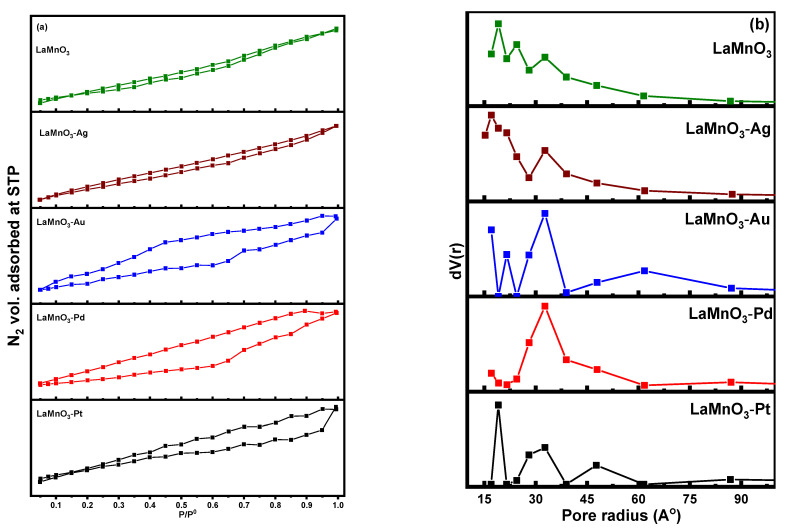
(**a**) N_2_ adsorption-desorption isotherms and (**b**) pore size distribution patterns of the samples.

The N_2_ adsorption-desorption isotherms and pore-size distribution patterns for synthesized samples are shown in Figure 5. As observed in the figure, all the samples are exhibiting the type IV isotherm with H3 type hysteresis loop, which is an indication that the samples possessed slit-shaped meso pores [36]. The shape of the hysteresis loop changed after the deposition of noble metals. The area of the hysteresis loop showed a distinct increase in the case of Pd, P, and Au deposited samples while it is decreased in the case of Ag deposited sample.

The textural properties of the samples, including the BET surface area, the average pore diameter and pore volume, are tabulated in Table 1. The BET surface area and pore volume values were highest for Pt-LaMnO_3_ sample. The least BET surface area was observed for the Ag-LaMnO_3_ sample, and the least pore size was that of LaMnO_3_ sample. The highest pore diameter was that of Pt-LaMnO_3_ and Au-LaMnO_3_ samples, closely followed by the Pd-LaMnO_3_ sample. The samples synthesized in this study exhibited BET surface area in the range of 12.2–20.1 m^2^/g, as shown in Table 1. These values are considerably high compared to those that have been attained by other methods of preparing perovskites (1–11 m^2^/g) [37]. The high surface area of the metal nanoparticles and through-channels of LaMnO_3_ become obvious, due to the removal of organic molecules during the thermal treatment. Additionally, there is an increase in the surface area from 14.6 to 20.1 m^2^/g when the Pt nanoparticles are deposited, this suggest that the dispersion of Pt on the LaMnO_3_ structure is high compared to Ag, Au and Pd metal particles, which has helped to enhance the surface area of the catalyst, hence improving the photocatalytic activity.

**Figure 6:**
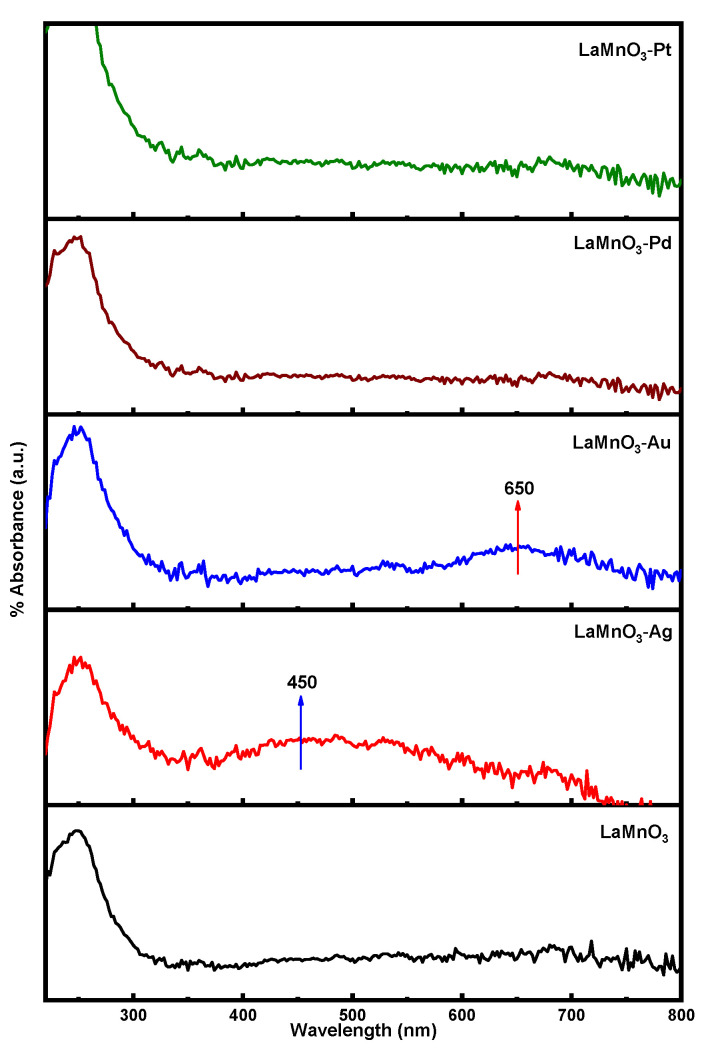
DR UV-vis spectra for the synthesized samples.

**Figure 7:**
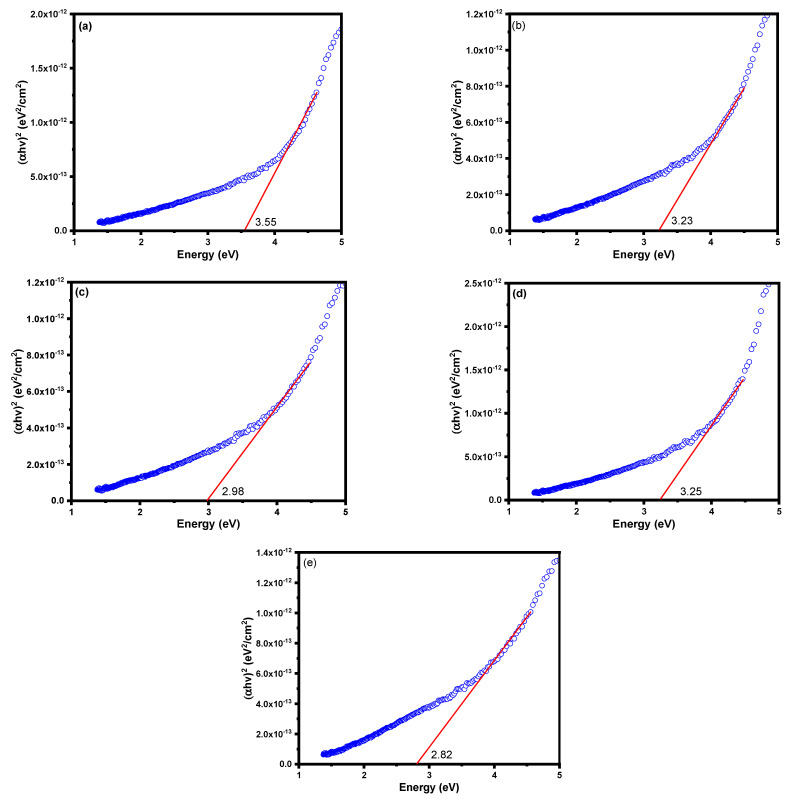
The Tauc plots for (**a**) LaMnO_3,_ (**b**) LaMnO_3_-Ag, (**c**) LaMnO_3_-Au, (**d**) LaMnO_3_-Pd, and (**e**) LaMnO_3_-Pt samples.

The DR UV-vis spectra of the bulk and noble metal deposited LnMnO_3_ samples are shown in Figure 6. The LnMnO_3_ sample exhibited absorption bands below 400 nm, which could be assigned to the charge transfer from valency band (VB) of O atoms to the conduction band (CB) of Ln atoms [38]. The DR UV-vis spectra of LnMnO_3_ deposited with noble metals exhibited an additional absorption band around 650 nm (visible region). The presence of Pt, Pd, Ag, and Au metal nanoparticles on the surface of LaMnO_3_ clearly resulted in appearance of absorption bands in the visible region. The excitation (charge transfer transition) of *4d* electrons of Pt/Ag/Au/Pd metal nanoparticles into the CB is responsible for the presence of visible absorption bands [39]. The bandgap energy values were calculated from the Tauc plots, as shown in Figure 7. The bandgap values for all the samples were obtained by drawing a tangent to the slope. The data revealed that the bandgap energy for bulk LnMnO_3_ sample (3.55 eV) is higher than that of Ag-LnMnO_3_ (3.23 eV), Au-LnMnO_3_ (2.98 eV), Pd-LnMnO_3_ (3.25 eV), and Pt-LnMnO_3_ (2.82 eV). The deposition of noble metals is expected to increase the particle size; hence, a decrease in the bandgap energy value is expected. This decrease in bandgap energy could alter the photocatalytic activity under visible light radiation [40].

**Figure 8:**
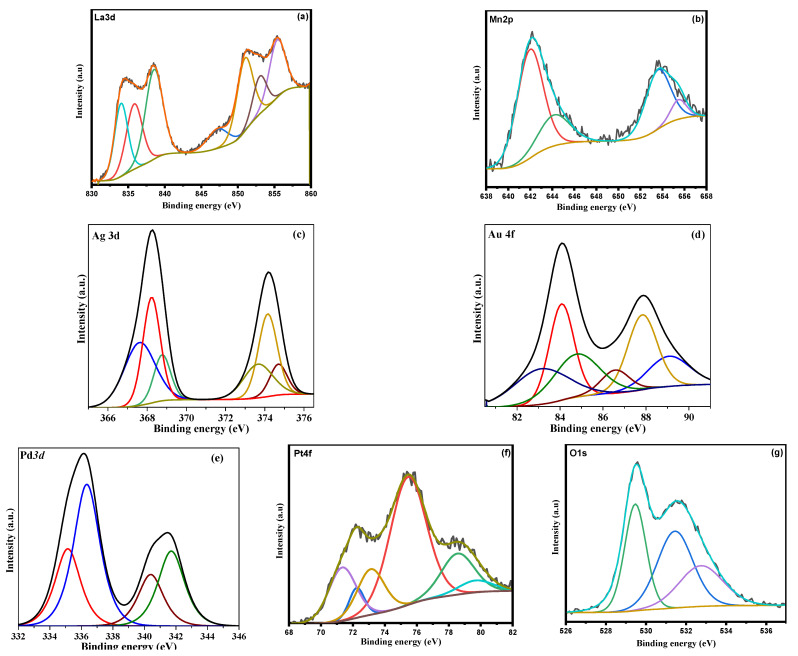
XPS spectra of (**a**) La3d (**b**) Mn2p, (**c**) Ag3d (**d**) Au4f (**e**) Pd3d (d), (**f**) Pt4f and (**g**) O1s.

The XPS analysis for the synthesized samples was carried out to characterize the surface electronic properties of the samples. Figure 8 shows the representative deconvoluted XPS spectra of the samples. It is known that La *3d* XPS peaks (Figure 8a) consist of La *3d_3/2_* and La *3d_5/2_* contributions and each spin orbit contribution have a doublet. The doublet appears due to the energy loss phenomenon induced by charge transfer from O *2p* to La *4f* [41]. The binding energies were appeared the range of 834–840 eV for La *3d_3/2_* contribution, which could be ascribed to La^3+^ species [42]. It is interesting to note that there is no considerable shift in the binding energies of La *3d* peaks in the case of noble metal deposited LaMnO_3_ samples, indicating that noble metals were not incorporated into the LaMnO_3_ structure.

As shown in Figure 8b, the Mn *2p_3/2_* signal could be deconvoluted into two components with a binding energy of 642.2 and 644.2 eV, which could be assigned to Mn^3+^ ions and Mn^4+^ species, respectively, [43]. Similar spectra were observed in all the synthesized samples; therefore, the Mn could have existed in both Mn^3+^ and Mn^4+^ in noble metal deposited LaMnO_3_ samples. Previously, Chen et al. [44] reported that Mn^3+^ could be partially oxidized into Mn^4+^ on the catalyst surfaces, which could produce structural defects. It is possible that a similar phenomenon could have occurred in the synthesized LaMnO_3_ materials in this study. It is possible to distinguish the Mn state precisely by calculating the difference between Mn *3s* peak and its satellite shake-up. The difference appeared to be around 5.3 eV, indicating that Mn is mainly presented in 3+ state in all the synthesized samples. It was previously reported that the Ag 3*d*_5/2_ peaks for surface bulk Ag^0^, Ag^+^, and Ag^2+^ species appear at 365.9, 366.4, and 367.1 eV, respectively [45]. However, in the present sample the Ag *3d_5/2_* peaks appeared at 367.6, 368.2 and 368.8 eV for metallic (Ag^0^) and oxidized (Ag^+^ and Ag^2+^) species, which are interacted with the LaMnO_3_ support. It is well known that a shift in binding energy occurs when surface species are interacted with the support. The Au *4f* XPS spectrum of the Au-LaMnO_3_ sample shows two peaks related to the core-level Au *4f_7/2_* and Au *4f_5/2_* contributions, further deconvoluted each into three different peaks corresponding to three different Au species 83.2, 84.1 and 85.0 eV. It was previously reported that XPS peaks for metallic Au species appear in the range of 82.9–84.5 eV, while the peaks at 85.8 and 86.5 eV could be assigned to the oxidized Au species (Au^+^ & Au^3+^) [46]. Therefore, the two peaks at 83.2 and 84.1 eV that appeared in the sample could be assigned to metallic Au species interacted with LaMnO_3_ semiconductor, while the third peak at 85.0 eV could be attributed to the oxidized Au species. The deconvoluted Pd *3d* XPS spectrum of the sample clearly shows the presence of four peaks at 336.4, 341.7, 335.1 and 340.4 eV, which could be assigned to Pd *3d_5/2_* and Pd *3d_3/2_* contributions of Pd^2+^ and Pd^0^ species, respectively, [47]. The percentage of peak areas of the Pd *3d* states clearly indicates that the contribution for Pd^2+^ species is more compared to Pd^0^ species in the synthesized sample. Considering the general mechanism of deposition of recued noble metal species on a support, in which the noble metal precursor first gets hydrolyzed and subsequently reduced and deposited on the surface of the support, it is possible that part of the hydrolyzed noble metal species gets oxidized during the thermal treatment and remains as an oxidized form on the surface of the sample. The O *1s* spectra of the samples could be deconvoluted into three different components with binding energy of 529.4, 531.4 and 533 eV, corresponding to lattice oxygen, adsorbed oxygen, and adsorbed H_2_O species, respectively [48].

### 3.2. Photocatalytic Activity of Bulk and Noble Metal Deposited LaMnO_3_ Samples

**Figure 9:**
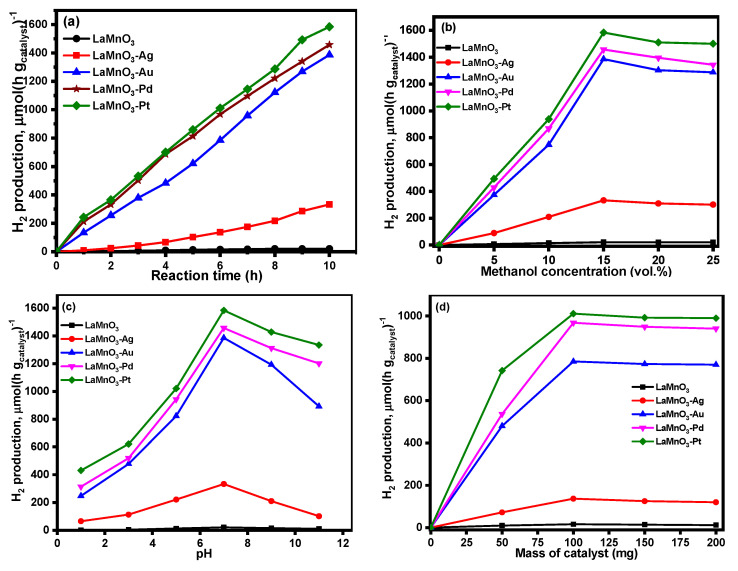
Photocatalytic H_2_ production, influence of (**a**) reaction time, (**b**) methanol concentration, (**c**) pH, and (**d**) mass of catalyst.

Figure 9a shows the influence of reaction time on photocatalytic H_2_ production over synthesized LaMnO_3_ catalysts. The bulk LaMnO_3_ catalyst showed no photocatalytic H_2_ production under visible light irradiation. Of the noble metal deposited LaMnO_3_ catalysts, Ag-LaMnO_3_ showed the most negligible H_2_ production; however, the Au-LaMnO_3_, Pd-LaMnO_3_, and Pt-LaMnO_3_ samples exhibited significantly high photocatalytic H_2_ production as a function of reaction time. Figure 9b displays the effect of methanol concentration on the photocatalytic H_2_ production. The results showed a similar trend, as that observed in Figure 9a. A steady increase was observed up to 15 vol % of methanol, after which a slight decrease was observed in the case of all the noble metal deposited LaMnO_3_ catalysts.

Figure 9c provides the results related to the effect of pH on H_2_ production over the synthesized catalysts. As the pH of the methanol aqueous solution increases to 7, an exponential increase in H_2_ production activity was recorded; however, an increase of pH beyond 7 caused in decrease in the photocatalytic activity of all the noble metal LaMnO_3_ catalysts, confirming that the photo reforming activity of synthesized catalysts is influenced by the pH of the reaction mixture. Figure 9d provides the results from the study of the role of the mass of the catalyst on photocatalytic H_2_ production over all the synthesized LaMnO_3_ nanocomposite catalysts. As observed from the figure, when the catalyst mass is increased from 50 to 100 mg, a significant increase in photocatalytic methanol reformation activity was observed. However, with a further increase in catalyst mass, there is no significant change in the photocatalytic activity observed.

**Figure 10:**
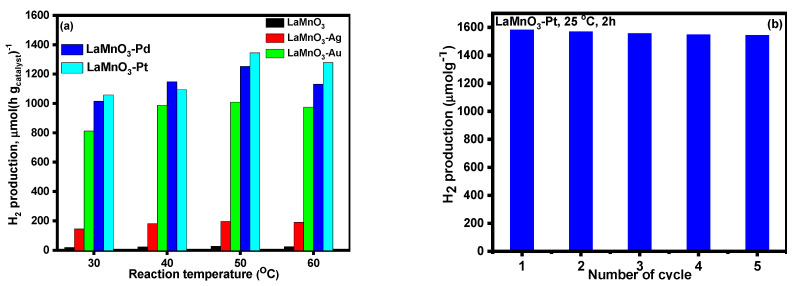
(**a**) Effect of reaction temperature on photocatalytic H_2_ production, (**b**) recyclability of the catalyst.

The influence of the reaction temperature on photocatalytic H_2_ production was studied and the obtained results are presented in Figure 10a. The results indicated that 50 °C is an ideal reaction temperature to obtain high H_2_ production. Up to 50 °C, a steady increase in H_2_ production was observed. With an increase in the reaction temperature (60 °C)., the reaction marginally slowed down. Korzhak et al. [49] studied photocatalytic H_2_ production over Cu-TiO_2_ nanocomposite catalysts at different reaction temperatures and observed that the reaction temperature has a considerable impact on the quantum yield. This is likely due to the thermal activation of product desorption. The observed decrease in H_2_ production beyond 50 °C may be caused by a decrease in the adsorption of methanol molecules on the catalyst surface. We have also reported a similar trend in the case of PtOx-TiO_2_ anatase nanomaterials [40]. Figure 10b provides the recyclability of the Pt-LaMnO_3_ catalyst. Specifically, the photocatalytic stability of the prototypical (highest active) Pt-LaMnO_3_ catalyst was revealed. Catalysts typically undergo photo-corrosion during the catalytic reaction; hence, it is widely known that investigating photocatalytic stability is important. As can be seen in Figure 10b, the Pt-LaMnO_3_ catalyst maintained its photocatalytic activity for H_2_ generation over an extended period. The structural stability and strong photo-corrosion resistance of the Pt system under the examined reaction conditions account for its recyclability [39].

With energy hv equal to or greater than the semiconductor bandgap, photon absorption starts photocatalytic reactions on semiconductors [50]. As a result, the photoinduced electrons move from the valence band (VB) to the conduction band (CB), resulting in the production of an electron-hole pair in the conduction band (CB). The resulting charge carriers can lead to the oxidation of electron donor species and the reduction of electron acceptor species, with the latter possessing a reduction potential that is higher in energy than the former. H^+^ ions serve as the electron acceptors in the photo-reforming reaction while the organic substrates, which are oxidized to CO_2_, serve as the electron donors. When organic molecules act as hole scavengers and undergo relatively quick and irreversible oxidation on the catalyst surface, the rate of photocatalytic H_2_ production is significantly accelerated [51]. Various nanocomposite materials, including ZnIn_2_S_4_-Au-TiO_2_ [52], Ag_2_O/Zn (O,S) Nanodiodes on Mesoporous SiO_2_ [53], ZnO/CdS hierarchical photocatalyst [54], Graphene oxide—Zn(O,S) photocatalyst [55] have been well established for their contribution to photocatalytic H_2_ evolution. The underlying mechanism in each case was somewhat the same; in the case of the noble metal deposited ZnO/CdS system, enhanced photocatalytic activity occurred because the heterostructure, not only facilitated an effective spatial separation of photo-induced electron-hole pairs, but also enhanced the redox ability of photocatalyst caused by an increase in redox potential. The work of the nanocomposites is to work towards effective spatial separation and enhanced redox ability.

## 4. Conclusions

In the present study, bulk LaMnO_3_ nanomaterial was synthesized by a combustion method using fine lemon powder as fuel. Novel green bio-reduction method was adopted for the deposition of various noble metals (Au, Ag, Pt, Pd) on the surface of LaMnO_3_. The characterization of the synthesized samples was accomplished by XRD, FT-IR, TEM, DR UV-*vis*, XPS, and N_2_ physisorption measurements. The existence of nanosized noble metal particles was observed from TEM image analysis. The deposition of noble metal led to improvement in visible-light absorption properties. The XPS results indicated the presence of both metallic and oxidized Ag, Au, Pd and Pt species on the surface of LaMnO_3_. The methanol reformation photocatalytic activity for bulk and noble metal deposited LaMnO_3_ samples was assessed. The results confirmed that the deposition of noble metals resulted substantial improvement in photocatalytic activity to H_2_ production. This enhancement is higher in case of Pt-LaMnO_3_ compared to other noble metal deposited samples. The increase in textural properties and the prevention of the recombination of photogenerated electrons and holes, thus, enhanced the photocatalytic activities of noble metal deposited LaMnO_3_ catalysts. The Pt deposited LaMnO_3_ catalyst has significant potential to produce H_2_ under visible light, which is both inexpensive and environmentally friendly, enabling the development of promises for H_2_ energy for the future.

## Figures and Tables

**Table 1:** Textural properties of the synthesized samples.

S. No.	Catalyst	BET Surface Area (m^2^g^−1^)	Pore Volume (cm^3^g^−1^)	Pore Diameter (Å)
1	LaMnO_3_	14.6	0.010	38
2	Ag@ LaMnO_3_	12.2	0.019	43
3	Au@ LaMnO_3_	13.5	0.015	64
4	Pd@ LaMnO_3_	12.4	0.018	61
5	Pt@ LaMnO_3_	20.1	0.023	64

## Data Availability

All data created is provided in this study.
